# Professionalism, professionalization, expertise and compassion: a qualitative study of medical residents

**DOI:** 10.1186/s12909-017-0864-9

**Published:** 2017-01-23

**Authors:** Susan P. Phillips, Nancy Dalgarno

**Affiliations:** 1 Department of Family Medicine, Queen’s University, PO Bag 888, 220 Bagot Street, Kingston, ON K7L 5E9 Canada; 2 Department of Family Medicine - Centre for Studies in Primary Care/Office of Health Sciences Education, Queen’s University, PO Bag 888, 220 Bagot Street, Kingston, ON K7L 5E9 Canada

**Keywords:** Empathy, Compassion, Professionalism, Professionalization, Expertise, Medical residency, Medical education

## Abstract

**Background:**

Formal and informal medical curricula convey expectations about professionalization, that is, the development of physician identity, and also about professionalism. This study examined whether, in general, junior residents experienced any dissonance between these roles and focused particularly on how they negotiated conflicts between compassion, self-care, duty and medical expertise.

**Methods:**

In 2015, purposive sampling was used to select 21 first-year residents at a Canadian medical school. Participants listened to a 5-min audio-recording narrated in either male or female voice. Facing compassion fatigue after three obstetrical disasters over less than 2 days the resident narrator asks to go home. Participants reacted in writing to questions about this request and relevant teaching/modelling. Responses were analyzed using a qualitative, exploratory, thematic research design.

**Results:**

Four themes were identified: i) empathy, self-doubt and fear of weakness, ii) the need for support from and communication with physicians and others, iii) education received, and iv) professionalization outranks professionalism. Participants agreed that under the circumstances the narrator’s care, compassion and request were appropriate. Never the less, many grappled with feeling that asking to be relieved of work demonstrated weakness and a shirking of responsibility. Respondents had received no formal teaching about balancing compassion for patients or self with professional duty. Preceptors’ informal teaching and modeling valorized scientific disengagement above all else. What emerged was participants’ drive to become detached clinicians who set aside emotional responses and interactions that could impede and be incompatible with professionalization. However, participants also recognized and lamented what was lost in such a transformation.

**Conclusion:**

In the transition from student to practitioner, trainees’ views and the modeling they receive shift emotion and compassion, whether for self or patients, from assets to liabilities as they aim to be invincible medical experts.

## Background

The excellent physician is seen to combine caring with the confidence and competence of medical expertise [1, 2]. However, these characteristics coexist in an uneasy and sometimes conflictual relationship, where caring is often an ‘extra’ to be enacted after the real work of expert clinician is completed. During training, tensions can arise from this divide between professionalism and professionalization. Constructs of professionalism encompass being an ethical, compassionate and virtuous person and doing medicine in a moral and competent manner [3]. In contrast, professionalization describes the process of entry into the profession of medicine, of identity formation via socialization and absorbing values that may be, but are not necessarily in keeping with professionalism [4, 5]. Professionalization is an ongoing process by which professionalism is attained through devotion to the profession and “acquiring a certain detachment and routinization towards one’s work; gaining formal knowledge and skills in order to make competent judgments; and developing a pretense of competence even though one may be privately uncertain” [6].

Students must negotiate dissonance they experience between professionalism and professionalization and, at times, must determine which will dominate. The cornerstone of professionalization is a confident, objective invincibility that may preclude, and always trumps caring and compassion [7, 8]. A presumed waning of caring and emotion with the rise of professional identity formation is the subject of much commentary [3, 9] informed by less research [9]. Professional identity formation has received attention in recent years [10, 11]. It has been framed as “a necessary foundation for professionalism” [12] but also as dynamic and evolving with changes in social norms and medical practices [10, 13]. Among studies of what happens as students become physicians, one demonstrated that the process of professional identity formation and of trying to balance competence and caring encouraged trainees to marginalize relationships, emotions and attitudes in favor of decisive and definitive actions and solutions [9]. Another found that students’ words valued humbleness while their actions valorized being the best and the brightest [14]. The students in that study suggested that patients’ emotional needs should be delegated to nurses, defining the physician’s role as medical expert. By deputizing caring to nurses, participants reinforced both the hierarchy of profession and of professional roles.

And what about learners’ emotional responses to their patients and the traumas they often witness? The ability to imagine, understand and resonate emotionally with the feelings of someone else is described as essential to the practice of medicine [15, 16]. An empathic physician enhances doctor-patient communication, and patient compliance, satisfaction and outcomes [16–20]. The strength of evidence is such that educational frameworks like CanMEDS [2] (an internationally used set of educational and practice competencies physicians require to effectively meet patient needs) enshrine the value of empathy in multiple roles including communicator, collaborator (where situational awareness is emphasized) and professional, with its focus on altruism, self-care and compassion [2]. Closely aligned with empathy is compassion, “a deep feeling of connectedness with the experience of human suffering that requires personal knowing of the suffering of others, evokes a moral response to the recognized suffering that results in caring that brings comfort to the sufferer” [21], and the ability to relate to the vulnerability of others in a meaningful way [18]. Contemporary medical pedagogy generally includes concepts of professionalization, emotional well-being, and clinical empathy [22]. Verghese, for example, purports that the connection essential to providing compassionate care arises from attending to patients’ physical and psychological well-being through listening and touch at the bedside [23]. However, without self-compassion or self-kindness the difficulty of bearing the suffering of others often produces compassion fatigue and less effective medical care, as physicians retreat physically and emotionally from patients, retaining only the role of medical expert [24, 25].

It would be erroneous to assume that valuing empathy or compassion for oneself or patients is embedded in the socialization underlying professional identity formation just because they are avowed in formal curricula [26]. How, then, do medical residents perceive empathy and compassion, and balance these with the model of dispassionate yet dutiful caregiver?

## Method

### Aim

Our aim was to study whether there is dissonance between professional identity formation and the caring and compassion inherent in professionalism, and specifically to:

1. identify what medical residents have learned explicitly and implicitly about balancing the emotions that arise during practice with personal and clinical obligations and limitations,
2. determine whether this teaching aligns with residents own values,
3. explore their responses to any dissonance between pedagogical messages and personal perceptions, and
4. examine whether any of the above vary between men and women.

### Design

We adopted an exploratory, qualitative design. To determine participants’ perspectives on the evolution of care during the transition from learner to physician [27, 28].

### Participants and Setting

We sent email invitations to all 100 Postgraduate Year 1 (PGYI) residents toward the end of their first year at one medium-sized medical school in Canada. Using purposive sampling, 21 participants were selected to ensure an overall balance of gender (representative of the female to male ratios in residency, overall) and specialty. Residents were, therefore, women and men (see Table 1), from multiple specialties and were graduates of many different medical schools. The two groups were of slightly different size as two residents who had been pre-allocated to Group 2 were unable to attend. Enrolment, never the less, allowed us to reach saturation (the point where no new information is obtained), hence, no further participants were recruited.

**Table 1:** Participants by group and gender

Group	Gender	
	Men	Women
1 (Female voice)	4	8
2 (Male voice)	3	6

### Description of research

In one of two simultaneous groups, medical residents listened to a 5-min audio recording of a resident recounting a harrowing 2 days doing obstetrics. The narrator, whose voice was altered to depict a woman for Group 1 and a man for Group 2 (see Table 1), described having clinical responsibility for a series of patients who experienced medical disasters (maternal death, neonatal death, life threatening complication) and eventually stated, “I cannot go on and must go home”. Individuals in each group responded to predetermined questions about how they and their preceptors would react in the situations described (see Table 2). All responses were confidential, written, and anonymous. Participants could withdraw at any time by choosing to not answer questions.

**Table 2:** Open-ended questions asked following the narrative

1. Summarize what happens in this story in a sentence or two.	
2. The resident eventually says “I can’t continue”. What do you think of this?	
3. What do you think the resident should have done?	
4. How would you have felt if you had been the resident?	
5. In your experience (as an MD, not a patient) how would preceptors have interacted with you: a. After the first disaster (maternal death)? b. After the second disaster (death of the baby)? c. After the telephone consult with neurosurgery? d. When the resident decides it is unsafe to be working?	
6. How do you think preceptors should have reacted? a. After the first disaster (maternal death)? b. After the second disaster (death of the baby)? c. After the telephone consult with neurosurgery? d. When the resident decides it is unsafe to be working?	
7. What guidance/teaching have you received regarding: a. Balancing care responsibilities with sensitivity to and distress about what happens in practice? b. Self-care (e.g., recognizing limits to your capacity, and when and whether it’s okay to say “I’ve reached my limit”)	

### Data analysis

We transcribed written responses into electronic form and then uploaded these to qualitative data analysis software (NVivo 10) for coding. An inductive, thematic design formed the qualitative framework for analyzing data. Three phases of the inductive analysis process were used to allow topics, categories and themes to emerge from the data. We coded responses into 94 topics that identified the subject of the data, grouped these into 12 broader categories that represented the meaning attached to connected topics and this resulted in four main themes that identified the relationships that emerged from the repetition among the categories (see Results) [29]. The themes were then compared and contrasted to relevant literature. To avoid individual biases an interactive and reflective process occurred between both researchers throughout analyses.

## Results

Responses of the 21 participants were consistent, concepts were repeated, and themes were common for women and men and regardless of the narrator’s gender. In general, participants’ faces showed distress while listening to the recording. Their written comments were long, detailed and thoughtful. No one opted out during the study and several thanked the researchers for initiating a discussion that had not occurred during their training programs.

Four distinct themes emerged: a) role conflict: empathy, self-doubt and fear of weakness, b) the need for support from preceptors, colleagues and friends, c) education received d) professionalization outranks professionalism. The quotes in this section are identified by the gender of the participant, the interview number and the group. For example, a male assigned the pseudonym ‘19’ who was a member of Group 1 is identified as (M19 G1).

- Role conflict: Empathy, self-doubt and weakness
  Most participants believed that the residents statement, “I can’t continue”, was an appropriate reaction in the circumstances described. They understood the narrator’s emotional exhaustion and viewed going home early as necessary to ensure patient and personal safety. As one respondent wrote: “I think given the extremely trying circumstances it was reasonable. I likely would have done the same thing. When one is so emotionally and physically drained, it is hard to continue to provide quality care to other patients” (M19 G1).
  A few of the participants imagined the emotional state of the narrator, expressed empathy, and, in a sense, validated the speaker’s mindfulness and self-compassion.
  “I completely empathize with him. I can place myself in his shoes and have been in similar positions where I am emotionally overwhelmed by dealing with difficult situations and patient deaths, with no support. I have been exactly where he has been and said to my senior resident that I cannot continue and need a break to pull myself together.” (F6 G2)
  However, when participants placed themselves in the narrator’s position they also felt overwhelmed, incompetent, and uncertain about future career capabilities in the face of such self-doubt and emotional collapse. Their statements included, “I likely would have felt very worried that I had done an inadequate job or that if I had done something different the situation could have ended differently” (M19 G1), and “I would have felt ashamed, devastated, unsupported, and like my career was in jeopardy. I would have catastrophized that no attendings would ever trust me again” (F5 G2).
  Equally evident was participants’ confusion about needing to block compassion so as to carry on with professional duty despite being overwhelmed emotionally. One resident stated: “I always feel that there's no duty assigned to me that I shouldn’t be able to do, with the proper support” (M9 G2), while another was, “uncertain what is best for patient care—to leave or stay” (F10 G2).
- Need for support
  All respondents believed the resident behaved responsibly and appropriately throughout. Both the medical care provided and the decision to withdraw from providing care were supported. For example, one participant wrote, “As residents, we are often our worst critics. If we decide that we cannot be working, that should be taken very supportively. This is the birth of burnout, depression, etc.” (F7 G1).
  All respondents also identified the benefit of debriefing with preceptors, team members, and/or friends and family, as each disaster unfolded. The following quotes highlight this:
  “…the responsibility to ensure that this (debrief) happened was with the attending.” (F6 G2)
  “Debrief/Discuss what had happened, how we did our best and that there are outcomes beyond our control.” (M19 G1)
  “I would have wanted to talk to my close friends who are also doing residency and could provide encouragement and support. I would have liked to talk to an attending physician I was close with to ask questions (how do you talk to someone who has lost a child, what could we have done differently, etc.) I would have liked time to process everything that had happened and to reflect on the experience.” (F8 G2)
  Most participants sought a debriefing that was primarily about how to manage clinical emergencies better. However, they also believed that a secondary purpose would be to address the resident’s emotional fallout. A few thought the resident should seek individual counselling. No participant reflected upon whether suggesting such counseling might imply that the narrator’s emotions and behaviors were personal problems rather than normal situational responses.
  Participants clearly articulated that any support received would be highly preceptor-dependent:
  “They (supervisors) would be preoccupied with their own emotions and worries. They wouldn’t know what to say to me. Some would be helpful, but most wouldn’t know how.” (F14 G1)
  “I have experienced thoughtful discussion and reflection and one occurrence of an attending throwing a death certificate at me and to fill it out without talking about it.” (M13 G1)
- Education received
  Very few participants had received formal teaching about managing caregiver distress in the context of patient suffering. Occasionally, informal advice was offered tangentially at Grand Rounds or during sessions on resident wellness. More frequently, however, participants learned from positive and negative role modeling:
  “It was not a direct teaching, but my preceptor discusses cases he found left him feeling off or unsettled and that makes it easier to bring up my own reactions to cases for an informal debrief.” (F14 G1)
  “I've even been burnt out and sort of figured it out on my own. I’ve been told ‘you should balance your clinic’ and ‘you should be self-aware’, but nothing tangible for when it actually happens, especially in residency where I have very little control.” (M2 G2)
  When asked about guidance and/or teaching regarding self-care, again almost all residents in the study reported receiving very little or no formal training. Without such education to offset or moderate role-modeling, residents assumed that the medical environment was one where self-care should always be subordinated to duty:
  “The ‘macho’ culture still persists. If you ‘reach your limit’ you are weak. I would worry that saying something like that would have professional consequences, e.g. being excluded from tough cases in the future or being given less clinical responsibilities because I couldn’t handle it.” (F15 G1)
  “I have learned about self-care and taking care of my own emotional well-being, but it is always in the context of what to do after work or on a post-call day, thus implying that it was not OK to reach my limit before the shift is complete.” (F10 G2)
- Professionalization outranks professionalism: real doctors soldier on
  As documented above, study participants overwhelmingly supported the narrator’s decision to leave. They wished that someone with authority had (i) intervened with patients, (ii) lauded the clinical acumen of the resident narrator, and (iii) offered some debriefing and support. It was when responding to questions about how they would have felt in the same situation that participants’ uncertainties surfaced, lodged in the gap between what is espoused (professionalism) and what is done (professional roles). Two participants explicitly described this conflict:
  “There is an expectation to continue working no matter what, otherwise you are seen as weak” (F15 G1).
  “The hidden curriculum is that we must have no limit. The spoken curriculum is to be aware of these limits and respect them. It is hard to respect them when there is nobody to do the work if we are not able to.” (F7 G1)
  Participants indirectly identified the interplay between the multiple roles clinical preceptors expect of trainees; first and foremost was being an expert. Juggling professional responsibility with compassion for self and patients introduced a complexity about which the implicit message they received was to ‘soldier on’:
  “I have had very few (preceptors) initiate a conversation about distress in practice Most emphasize the importance of patient care. The fact that this aspect (patient care responsibilities) is addressed much more consistently has engrained in me this idea that my responsibility trumps any distress I may be experiencing.” (F10 G2)

## Discussion

Participants’ non-verbal and written communication during this study spoke to a humanity and sensitivity for both the patients whose obstetrical disasters were described in the narrative and the narrator’s gradual unraveling of confidence and caregiving capacity. Respondents’ compartmentalized person and profession; as persons they reacted with empathy and compassion but their physician-selves focused on duty, training expectations and the onward march of the invincible, scientific professional. Many grappled with whether this need to compartmentalize arose from within or was external and systemic. Participants concluded that regardless, to fit in to the profession they must detach from emotions and compassion, and become objective experts.

Professional status, empathy and compassion were all in flux among study participants. They retained their emotional awareness of patients, colleagues and self but in keeping with Doja et al’s findings [30], were beginning to see these as liabilities and barriers to entry into the profession. Participants’ still valued their inherent compassion yet a sense of emerging professionalization created fear that giving in to emotions would detract from their real work. Although respondents all thought the narrator did nothing wrong, faced with the same situation they would have felt ashamed or inadequate for succumbing to emotions and being unable to continue. They had neither the time, models, nor self-confidence to oppose the dominant paradigm of professionalization and to instead merge medical expert, their perceived primary role, with the caring, comforting and self-compassion of professionalism.

In the transition from compassionate resident to competent independent physician, empathy and emotional intelligence may be the casualties [30, 31]. The residents in our study felt the need to sacrifice compassion for self and patients to unwavering duty. However, perhaps because they were still early in their residencies, the tendency to be empathetic kept surfacing and conflicting with their image of excellence—the invincible, dutiful doctor.

The evidence that being a caring person is an essential characteristic of the excellent physician is clear [16, 19]. Most formal medical curricula acknowledge this and encourage the expression of empathy and compassion and the self-awareness to be comfortable with such expression [2]. Objectives Canadian students must meet to be licensed as physicians echo the importance of these characteristics [32]. North American postgraduate accreditation guidelines state, “The program must be able to demonstrate that residents exhibit integrity, honesty and compassion in the delivery of the highest quality care” [33].

On the other hand, dissonance between what educational institutions articulate explicitly and what teachers and supervisors model, gives rise to very powerful informal and hidden curricular imperatives [34]. To carry out the main task physicians assign themselves - to cure - participants observed the tendency of preceptors to retreat to objectivity and detachment [5]. Implicit messages from faculty positioned the objective and detached role of medical expert ahead of more elusive characteristics like empathy and compassion. Some participants’ perceptions were that these ‘soft’ behaviors are for after evidence-based medicine has happened. The conflict between this ‘real work’ and compassion and self-care gave rise to a confused view of professionalism. Empathy and compassion had the potential to disable the dispassion and objectivity many found to be so fundamental to professional identity. For them, absenting oneself even briefly when overwhelmed by emotion demonstrated a breach of professionalism and the duty to care. The difficult challenge with respect to addressing professional anxiety and distress seemed to be finding a way to behave objectively and with appropriate detachment while maintaining empathy and compassion.

The majority of respondents perceived that balancing compassionate care and objective duty was difficult for faculty as well, who were seen to escape into emotional neutralization (e.g., F21 G1, M2 G2). This abandonment of emotional involvement has also been identified by others as a stage that trainees implicitly recognize and that they must pass through to become effective physicians [30]. Without consciously deciding, our participants, all first-year residents, may be suppressing empathy and compassion because these characteristics compete with and divert from professionalization. And so, as a way to minimize stress in the face of very sick patients and become excellent physicians like their teachers, who can soldier on, are unshakeable and never give up or give in to emotion, residents try to distance themselves from empathy and compassion. Potentially they become doctors who are afraid to care or feel the self-compassion that mitigates compassion fatigue. Our study suggests that perhaps we as preceptors could do better modeling of how to integrate professionalism, empathy, compassion and professionalization in ways that work for physicians and patients.

Our findings are limited to the perceptions of a sample of residents at one university. However, prior to residency participants attended many different medical schools and therefore bring experiences from multiple settings to the research. To broaden understanding, ethnographic methods using qualitative research involving students, physicians, patients and family members could be conducted. The similar responses by women and men, and to a male or female narrator might be different among a larger sample.

## Conclusion

In moving from student to resident to practitioner medical trainees learn professionalism and experience professionalization. For participants in this study the explicit importance of self-awareness, compassion, empathy and caring inherent in professionalism is undermined and eroded by the more immediate and powerful modeling of professional identity formation they observe and absorb. Although junior residents retain awareness of their emotional responses to patients’ crises, these are seen as liabilities to succeeding at the primary enterprise of medicine—medical expertise. The impact on patients of abandoning empathy and compassion on the journey to becoming a doctor is presently not known and can only be imagined.
